# Quantitative analysis of the tomato nuclear proteome during *Phytophthora capsici* infection unveils regulators of immunity

**DOI:** 10.1111/nph.14540

**Published:** 2017-04-10

**Authors:** Andrew J. M. Howden, Remco Stam, Victor Martinez Heredia, Graham B. Motion, Sara ten Have, Kelly Hodge, Tiago M. Marques Monteiro Amaro, Edgar Huitema

**Affiliations:** ^1^ Division of Plant Science School of Life Sciences University of Dundee at the James Hutton Institute (JHI) Invergowrie Dundee DD2 5DA UK; ^2^ Dundee Effector Consortium JHI Invergowrie Dundee DD2 5DA UK; ^3^ Section of Population Genetics Technische Universität München 85354 Freising Germany; ^4^ Cell and Molecular Sciences JHI Invergowrie Dundee DD2 5DA UK; ^5^ Wellcome Trust Centre for Gene Regulation and Expression School of Life Sciences University of Dundee Dow Street Dundee DD1 5EH UK

**Keywords:** immunity, nucleus, *Phytophthora*, plant–microbe interactions, quantitative proteomics, tomato

## Abstract

- Plant–pathogen interactions are complex associations driven by the interplay of host and microbe‐encoded factors. With secreted pathogen proteins (effectors) and immune signalling components found in the plant nucleus, this compartment is a battleground where susceptibility is specified. We hypothesized that, by defining changes in the nuclear proteome during infection, we can pinpoint vital components required for immunity or susceptibility.
- We tested this hypothesis by documenting dynamic changes in the tomato (*Solanum lycopersicum*) nuclear proteome during infection by the oomycete pathogen *Phytophthora capsici*. We enriched nuclei from infected and noninfected tissues and quantitatively assessed changes in the nuclear proteome. We then tested the role of candidate regulators in immunity through functional assays.
- We demonstrated that the host nuclear proteome dynamically changes during *P. capsici* infection. We observed that known nuclear immunity factors were differentially expressed and, based on this observation, selected a set of candidate regulators that we successfully implicated in immunity to *P. capsici*.
- Our work exemplifies a powerful strategy to gain rapid insight into important nuclear processes that underpin complex crop traits such as resistance. We have identified a large set of candidate nuclear factors that may underpin immunity to pathogens in crops.

## Introduction

Plant−microbe interactions are complex and feature signalling exchanges between host and pathogen. Plants have evolved a sophisticated immune system that operates through perception of pathogen‐ or microbe‐associated molecular patterns (PAMPs/MAMPs) resulting in pattern triggered immunity (PTI) (Jones & Dangl, 2006). PAMP signalling drives transcriptional reprogramming, activating gene expression associated with defence (Eulgem, 2005). PTI‐induced nuclear signalling thus demands the trafficking of proteins between the cytoplasm and nucleus. Plant disease resistance proteins (Deslandes *et al*., 2003), mitogen activated protein kinase signalling components and transcription factors are thought to act in such a fashion while regulating transcriptional responses (reviewed by Rivas (2012) and Shen & Schulze‐Lefert (2007)). The nuclear proteome therefore is dynamic, regulated and adaptable to external cues. The exact changes that take place in this compartment during plant−microbe interactions, however, are unknown.

Pathogens must compromise structural barriers and suppress host immune responses to promote growth. In addition to enzymes that degrade or detoxify structural and chemical barriers, pathogens secrete proteins, termed effectors, to the apoplast (apoplastic effectors) and into the host cell cytoplasm (cytoplasmic effectors) to suppress immunity (Schornack *et al*., 2009). Genome sequence analyses and functional and cell biological approaches have helped identify a multitude of cytoplasmic effectors that target distinct cellular compartments (Kamoun, 2006). These efforts have led to the general view that pathogen effectors are the principal determinants of field epidemics.

Contemporary conceptual models assume that, upon delivery to their intended host cellular address, effectors bind their target(s) with the aim of modifying or perturbing processes required for immunity or pathogen growth (Jones & Dangl, 2006; Mukhtar *et al*., 2011). Large numbers of nuclear targeting pathogen effectors have been identified, including the *Pseudomonas* outer protein P2 effector from *Ralstonia solanacearum* (Deslandes *et al*., 2003; Tasset *et al*., 2010), *Xanthomonas* outer protein D and AvrBs3 from *Xanthomonas campestris* pv *vesicatoria* (Hotson *et al*., 2003; Kay *et al*., 2007; Canonne *et al*., 2011), and HsvG and host‐specific virulence G and B respectively from *Pantoea agglomerans* pv *gypsophilae* (Nissan *et al*., 2006; Weinthal *et al*., 2011). Along with the identification of nuclear effectors, progress in dissecting nuclear signalling pathways has been made (reviewed in Canonne & Rivas, 2012; Rivas, 2012; Motion *et al*., 2015). However, a detailed understanding of the changes that occur to the host nuclear proteome in response to infection is missing. Given the large effector repertoires of many plant pathogens and a vast set of host nuclear factors with roles in immunity, the application of a global approach that allows examination of all changes may provide a valuable overview of infection‐associated processes. While numerous studies have examined pathogen‐induced transcriptional changes in plants (reviewed by Eulgem (2005) and Katagiri (2004)), these studies do not provide information on changes that occur to the proteome. In addition, gene expression does not provide information about protein abundance, localization and post‐translational modification. Quantitative proteomics thus allows the assessment of changes in the protein complement of the cell, providing additional information on cellular processes.

Here, we have assessed changes in the tomato (*Solanum lycopersicum*) nuclear proteome during infection by the oomycete pathogen *Phytophthora capsici*. Previously, we showed that *P. capsici* forms haustoria in the early stages of infection and that substantial changes in gene expression take place at 8 h (early biotrophy) and 24 h (late biotrophy) after inoculation (Jupe *et al*., 2013). We took advantage of our ability to isolate tomato nuclei from infected (living) leaf tissue to combine a nuclear enrichment method with liquid chromatography−tandem mass spectrometry (LC‐MSMS) technology to survey and quantify changes in the nuclear proteome upon *P. capsici* infection. Our results point to dramatic changes in the host nuclear proteome, affecting processes such as DNA binding, protein binding and oxidoreductase activity, and involving factors with known roles in immunity. Based on our analyses, we selected members of the AT‐Hook‐Like (AHL) protein family and found that a subset contribute to immunity. Two of these AHL proteins were found to accelerate PTI responses, suggesting that modification of AHL protein abundance or function could be used to enhance disease resistance. Our work demonstrates the power of quantitative proteomics to probe and understand host processes during pathogen infection by identifying proteins with roles in immunity. We suggest that our approach is ideally suited to quickly gaining important insights into plants with sequenced genomes.

## Materials and Methods

### Growth of *Solanum lycopersicum*,* Nicotiana benthamiana* and *Phytophthora capsici*

*Solanum lycopersicum* (cv Moneymaker) and *Nicotiana benthamiana* were grown in a glasshouse under 16 h of light and at *c*. 25°C : 22°C, day : night ± 2°C. *Phytophthora capsici* strain LT1534 was grown on V8 agar plates at 24°C in the dark for 3 d before being moved to a continuous light incubator at 24°C to stimulate sporangium formation.

### Tomato infections

To induce zoospore release, ice‐cold distilled water was poured onto *P. capsici* plates, and sporangia were dislodged with a glass spreader. Sporangial suspensions were collected and incubated in bright light conditions at room temperature to stimulate zoospore release. Spores were adjusted to 500 000 spores ml^−1^ for inoculations. The second and third leaves from 4‐wk‐old *S. lycopersicum* plants were detached and placed in square Petri dishes with damp tissue paper. Leaves were spray inoculated with *P. capsici* spores or with distilled water as a control. Inoculated leaves were incubated at 23°C under continuous light. Leaves were harvested after 8 and 24 h and flash frozen in liquid nitrogen before storage at −80°C until they were used for nuclear isolations. The experiment was carried out three times to generate independent biological replicates.

### Nuclear enrichment and protein purification

Frozen leaf tissue (40 g) was ground to a fine powder in liquid nitrogen and re‐suspended in 200 ml of ice‐cold nuclei isolation buffer (NIB) (25 mM piperazine‐N,N′‐bis(2‐ethanesulfonic acid) (PIPES), 10 mM NaCl, 5 mM ethylenediamine tetracetic acid (EDTA), 250 mM sucrose, 1% thiodiglycol, 0.15 mM spermine, 0.5 mM spermidine, 0.1% nonident P40, 0.2 mM phenylmethylsulfonyl fluoride and 1 × protease inhibitor (Thermo Scientific, Waltham, MA, USA), pH 7.0). Suspensions were filtered through two layers of Miracloth (cat no. 475855; Merck Millipore, Watford, UK) and then 500‐, 100‐ and 60‐μm filters (custom made). Filtered suspensions were centrifuged at 4650 ***g*** for 20 min at 4°C. The pellets were retained and gently re‐suspended in 25 ml of NIB before centrifugation at 1940*** g*** for 10 min at 4°C. Pellets were then re‐suspended in 15 ml of NIB and centrifuged at 1480 ***g*** for 10 min at 4°C. The resulting pellets were gently re‐suspended in 15 ml of NIB and centrifuged at 1480 ***g*** for 10 min at 4°C. Subsequently, pellets were re‐suspended in 15 ml of NIB and filtered through two layers of Miracloth before being centrifuged at 1480 ***g*** for 10 min at 4°C. The pellets obtained were then washed twice by suspending them in 25 ml of NIB and centrifuging at 1480 ***g*** for 10 min at 4°C. Finally, pellets were re‐suspended in 1 ml of NIB and transferred to a 1.5‐ml Eppendorf tube before being spun at 1500 ***g*** for 10 min at 4°C. The supernatant was removed and the pellet flash frozen in liquid nitrogen and stored at −80°C.

Proteins were isolated from nuclei by suspending pellets in 200 μl of protein extraction buffer (100 mM Tris (pH 7.5), 15 mM MgCl_2_, 7.5% glycerol, 1% SDS, 8 M urea, 5 mM DTT, 2 × protease inhibitor (Sigma), and 1% triton × 100). Suspensions were vortexed vigorously and placed on ice for 2 h while vortexing for 2 min every 20 min. The suspension was centrifuged at 12 470 ***g*** for 10 min at 4°C and the supernatants were kept. The protein extract was quantified using the bicinchoninic acid (BCA) protein assay reagent (Thermo Scientific) following the manufacturer's instructions.

### In‐gel fractionation, protein digestion and sample clean‐up

For each protein sample (8‐h noninfected, 8‐h infected, 24‐h noninfected and 24‐h infected), 50 μg was loaded onto pre‐cast 4–20% polyacrylamide gels (Bio‐Rad) and samples were run two‐thirds of the way down the gel. Each lane was cut into eight equally sized pieces and each section cut into 1‐mm^3^ cubes. Gel pieces were washed with 100 mM NH_4_HCO_3_ and acetonitrile before reduction and alkylation with 10 mM DTT and 55 mM iodoacetamide, respectively. Proteins were subject to in‐gel digestion with sequencing‐grade trypsin (Roche) and digestions were carried out at 37°C overnight. The resulting peptides were cleaned using a C18 (POROS R2; Applied Biosystems, Waltham, MA, USA) column. Clean peptide samples were dried down to *c*. 10 μl using a vacuum centrifuge before being re‐adjusted to a final volume of 30 μl with 0.1% trifluoroacetic acid.

### Mass spectrometry and data analysis

Peptide samples were analysed on an Orbitrap Velos (Thermo Scientific) mass spectrometer with a 156‐min gradient using the following buffer conditions: A = 0.1% formic acid; B = 80% acetonitrile and 0.1% formic acid. The gradient run consisted of the following steps: 2% B for 0–4 min, 2–40% B for 4–128 min, 40–98% B for 128–130 min, 98% B for 130–150 min, and 2% B for 151–156 min. The parent ion scan was set at a resolution of 60 000 while the MS/MS was set at ‘normal scan’ with a peak width of 0.6. Scans were undertaken at 335–1800 *m/z* and the MS/MS had a minimum signal of 5000 ions. The mass window tolerances were set to 10 ppm for all data‐dependent acquisition. The top 15 ions were selected for MS/MS, using collision‐induced dissociation (CID), on ions 2+ and over (1+ ions excluded) with a collision energy of 35 and an activation time of 10 ms. The dynamic exclusion repeat count was 1, with a repeat duration of 30 s, an exclusion list size of 500 and an exclusion duration of 45 s. A 5‐μl fraction of the 30‐μl peptide solution was loaded onto the instrument for each run. Raw MS data files were subject to a comprehensive analysis pipeline outlined in Supporting Information Fig. S1. Protein identification and quantification were carried out using maxquant software version 1.4.1.2 (Cox & Mann, 2008). Raw MS data files were searched against a combined *S. lycopersicum* (Tomato Genome, 2012) and *P. capsici* (Lamour *et al*., 2012) genome file (Notes S1) and quantification data were acquired using the label‐free quantification tool (LFQ) within maxquant (settings outlined in Notes S2) (Cox *et al*., 2014). Full details of how maxquant generates peak lists and determines false discovery rates are detailed in Cox & Mann (2008). The MS proteomics data have been deposited in the ProteomeXchange Consortium (Vizcaino *et al*., 2014) via the PRIDE partner repository with the data set identifier PXD003260.

Protein identification and quantification data were analysed in the Perseus software package (Tyanova *et al*., 2016) using two‐sample *t*‐tests with a false discovery rate (FDR) of 0.05 to identify proteins showing significant differences in expression. Proteins identified with at least two peptides (shared, unique or razor) were selected for downstream analysis. Proteins with a consistent presence/absence expression profile between treatments and across all biological replicates were identified using simple custom r scripts (Stam (2015) and summarized in Notes S3). To interrogate the data further, we used a simple pipeline on our local galaxy server (Goecks *et al*., 2010; Cock *et al*., 2013) to identify potential nuclear proteins within the protein identification data set. A detailed description, including scripts used and analysed data, can be found on the GitHub page (Stam, 2015). In short, amino acid sequences for those proteins identified by MS were extracted from the SOL genomics protein database v. ITAG2.4 (Tomato Genome, 2012). This data set was subjected to predictnls (default settings) (Cokol *et al*., 2000) and nlstradamus (two‐state model; posterior threshold 0.6) (Nguyen Ba *et al*., 2009) to identify proteins with nuclear localization signals, and the Nucleolar Localization Sequence Detector (nod) (default settings) (Scott *et al*., 2010, 2011) to identify proteins with nucleolar localization signals. Additionally, we used wolf psort (plant) (Horton *et al*., 2007) and filtered all proteins marked as nuclear. The results of these four searches were combined and duplicate IDs were removed.

### Cloning candidates for overexpression and EGFP fusion

Tomato AT‐Hook‐Like (AHL) coding genes were amplified using Phusion polymerase (Thermo Scientific) from cDNA generated from infected tomato material (detailed in the ‘Tomato infections’ section above). Forward primers used for amplification had CACC bases added to the 5′ end to allow cloning of amplicons into the Gateway entry vector pENTR‐D‐Topo (Life Technologies, Paisley, UK) following the manufacturer's instructions. Primer sequences are provided in Table S1. Verified entry clones were used for recombination into Gateway compatible destination vectors using the Gateway LR clonase II enzyme (Life Technologies) following the manufacturer's instructions. Genes were re‐combined into the 35S overexpression binary vector pB7WGF2 (Karimi *et al*., 2002) to create N‐terminal enhanced green fluorescent protein (EGFP) fusions. All clones were sequence verified before further use.

### Confocal microscopy

*Agrobacterium tumefaciens* strain AGL1 carrying respective EGFP fusion constructs were grown overnight at 28°C and 200 rpm. Cells were spun down and re‐suspended in infiltration buffer (25 mM MgCl_2_ and 15 μM acetosyringone) to a final optical density (OD) of 0.1. Four‐week‐old *N. benthamiana* plants expressing RFP‐labelled histone (nuclear marker) were infiltrated with the bacterial suspension and incubated for 48 h in the glasshouse before microscopy. Confocal imaging was carried out on a Zeiss LSM 710 microscope with a W Plan Apochromat ×40/1.0 DIC M27 water dipping lens using the following settings: EGFP (488 nm excitation and 495–534 nm emission); mRFP (561 nm excitation and 592–631 nm emission). Leaf discs were harvested from infiltrated plants at the time of imaging for protein extractions and western blotting with anti‐EGFP antibody to establish fusion protein stability.

### Western blotting

To assess histone enrichment within nuclear preparations, nuclear protein extracts and total leaf protein extracts were subject to western blotting with anti‐histone H3 antibody (Abcam, Cambridge, UK). To generate total leaf protein extracts, tissue was ground in liquid nitrogen and suspended in the protein extraction buffer used for the nuclear preparations. Protein concentrations were determined using the BCA assay (described in the ‘Nuclear enrichment and protein purification’ section, above) and equal quantities of nuclear protein extract and total leaf protein extract were loaded onto pre‐cast 4–20% polyacrylamide gels (Bio‐Rad) before transfer to PVDF membranes using the Trans Blot Turbo Transfer System (Bio‐Rad). Membranes were blocked for 30 min in TBS‐Tween (TBS‐T) with 5% milk before incubation overnight at 4°C with anti‐histone H3 antibody (1 : 2500) in TBS‐T plus 5% milk. Membranes were then washed for 5 × 5 min in TBS‐T before incubation for 1 h in anti‐rabbit IgG‐HRP (Santa Cruz Biotech, Dallas, TX, USA) (1 : 20 000) in TBS‐T plus 5% milk before being washed for 5 × 5 min in TBS‐T. Blots were incubated with Luminata Forte HRP substrate (Millipore) and imaged on a GBox TX4 Imager (Syngene, Camridge, UK). To determine the level of nonnuclear contamination within samples, gels were run as described earlier in this section and probed with an endoplasmic reticulum marker calnexin homologue 1/2 antibody (Agrisera, Vannas, Sweden) at a dilution of 1 : 1000 for the primary antibody and 1 : 10 000 for the secondary antibody anti‐rabbit IgG‐HRP. Blots were also performed with the cytoplasmic marker UDP‐glucose pyrophosphorylase (Agrisera) at dilutions of 1 : 1500 and 1 : 10 000 for the secondary antibody anti‐rabbit IgG‐HRP. Blots were washed and visualized as described earlier in this section.

To establish EGFP‐fusion construct stability, protein extracts were generated by grinding *c*. 100 mg of plant tissue in 200 μl of SDS loading buffer (0.25 M Tris, pH 6.8, 6% SDS, 40% glycerol, 0.04% bromophenol blue and 100 mM DTT), and then centrifuged for 10 min at 17 000 ***g***. Blots were generated as described in the previous paragraph and blocked for 30 min with 5% milk in TBS‐T, and incubated overnight with anti‐EGFP antibody (1 : 3000) (Cambio, Cambridge, UK). Blots were washed for 3 × 5 min in TBS‐T and were then incubated for 1 h with anti‐mouse HRP antibody (Santa Cruz Biotech) (1 : 20 000) in 5% milk in TBS‐T before being washed three times in TBS‐T for 5 min. All membranes were visualized as described in the previous paragraph.

### Overexpression of tomato proteins in *N. benthamiana* for infection and PTI assays

The second and third leaves from 5‐wk‐old *N. benthamiana* plants were infiltrated with *A. tumefaciens* cells carrying either pB7WGF2 (N‐terminal EGFP tag) overexpression constructs or empty vector pB7WGF2 for overexpression of free EGFP as a control. For infection assays, *A. tumefaciens* cells were prepared for infiltration as described in the ‘Confocal microscopy’ section and infiltrated at an OD of 0.1 for all constructs. Infiltrated plants were incubated in a glasshouse under 16 h of light and at 25°C : 22°C, day : night. After 48 h, leaves were detached and placed in Petri dishes with moist tissue paper. A 10‐μl drop of *P. capsici* zoospore suspension (generated as described in the ‘Tomato infections’ section and adjusted to a density of 50 000 spores ml^−1^) was placed next to the point of infiltration and leaves were incubated at 23°C in 16 h of light. *Phytophthora capsici* lesions were measured 48 and 72 h post‐infection. ANOVAs with Bonferroni‐corrected post hoc two‐sample *t*‐tests were carried out to identify significant changes in infection between constructs at an FDR of 0.05. For PTI assays, each construct was infiltrated at an OD of 0.1 as described in the ‘Confocal microscopy’ section. After 48 h, leaves were infiltrated with culture filtrate (CF), from *P. capsici* liquid culture, or pea broth (PB) medium as a control. Leaves were scored visually for cell death using a scale from 0 to 6 (with 0 being no death and 6 being confluent cell death), as described previously by Stam *et al*. (2013), 24, 48 and 72 h post‐CF treatment.

## Results

### A nuclear enrichment‐based strategy to quantify tomato nuclear proteins during *P. capsici* infection

**Figure 1:**
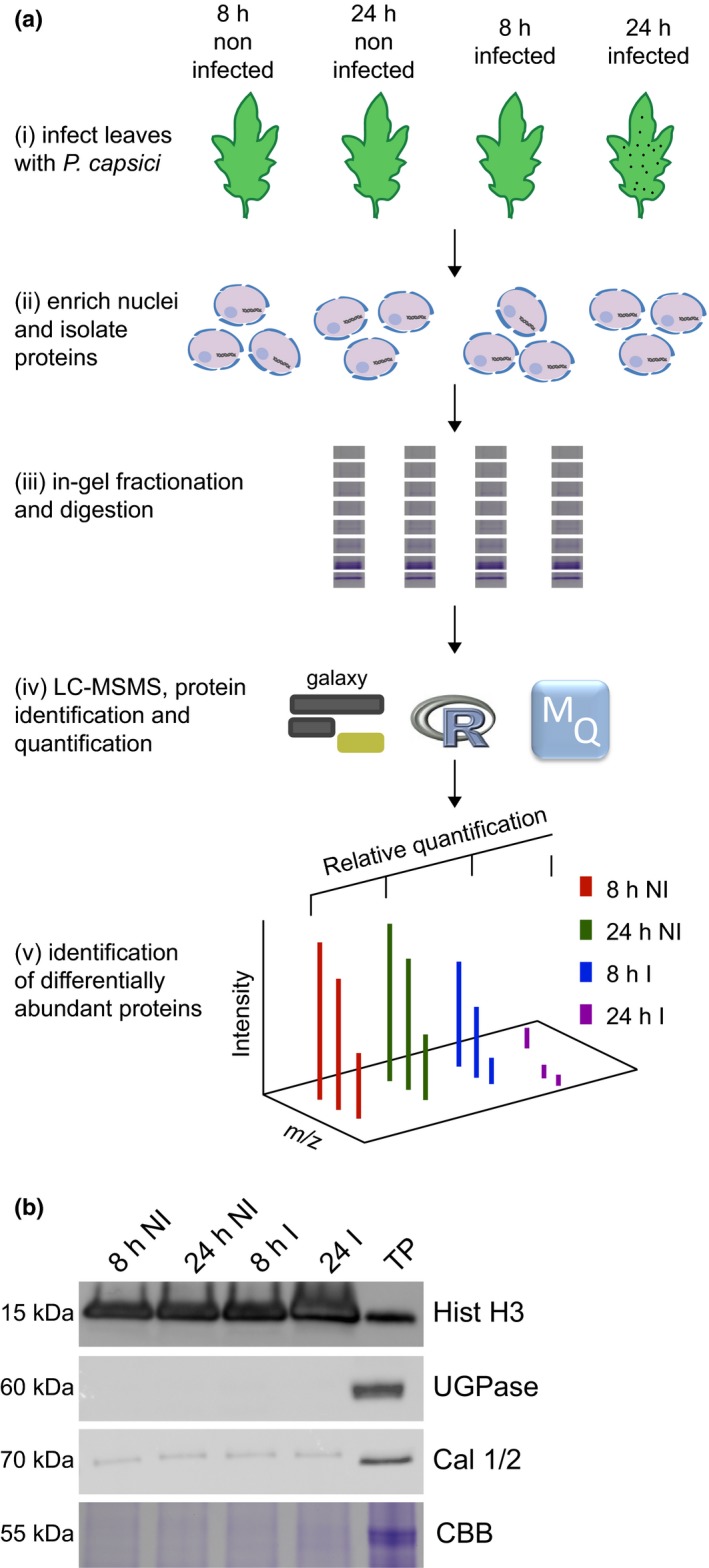
A simple workflow combining nuclear enrichment with quantitative mass spectrometry allows the study of host nuclear processes during infection. (a) Method overview. Detached leaves from 4‐wk‐old tomato plants were spray‐inoculated with *Phytophthora capsici* zoospores at a concentration of 500 000 spores ml^−1^, or water as a control (i). Leaves were harvested 8 and 24 h post‐infection and subject to nuclear enrichment (ii). Nuclear protein extracts were generated and fractionated in‐gel and digested with trypsin (iii). Peptide samples were subjected to liquid chromatography−tandem mass spectrometry (LC‐MSMS) analysis and peptide identification and label‐free quantification was carried out using the maxquant and perseus software packages, to identify proteins that were differentially expressed during infection. Nuclear predictions were performed using prediction software on our in‐house Galaxy server as described in the Methods. Subsequent data filtering was completed using the r software package (iv and v). Three independent biological replicates were generated. I, infected samples; NI, noninfected samples (v). (b) Successful enrichment of nuclear proteins demonstrated by western blotting with anti‐histone H3 antibody and subcellular markers. Protein extracts from enriched nuclear samples were compared to a total protein extract (TP) from tomato leaves. Protein concentrations were adjusted for equal loading. Nonnuclear contamination was assessed by probing with anti‐UDP‐glucose pyrophosphorylase (UGPase) antibody (cytoplasm) and calnexin homologue 1/2 antibody (endoplasmic reticulum). Samples were also run on gels and stained with Coomassie brilliant blue (CBB) to assess protein loading and Rubisco abundance. The figure shows the results from a single biological replicate. Blots for all three replicates are provided in Supporting Information Fig. S2.

We sought to quantify and assess dynamic changes that occur in the host nuclear proteome as a consequence of *P. capsici* infection. To reduce the abundance of cytosolic (contaminant) proteins in our experiments and to allow the identification of novel nuclear proteins, we devised a nuclear enrichment and protein extraction protocol, suited for quantitative MS (Fig. 1a). Nuclei were enriched from infected and mock‐infected tomato leaves at 8 and 24 h post infection and proteins were extracted before western blot and LC‐MSMS analyses. To assess nuclear enrichment, we probed extracts with anti‐histone H3 antibody and compared concentrations of histone H3 between enriched samples and a total leaf protein extract. Histone H3 was significantly enriched in nuclear samples relative to an equivalent total protein extract from leaves (Fig. 1b). Western blots with anti‐UDP‐glucose pyrophosphorylase (UGPase) antibody and an antibody against calnexin homologue 1/2 showed considerable depletion of cytosolic and endoplasmic reticulum (ER) contaminants in nuclear enriched samples relative to total leaf protein extracts (Fig. 1b). Coomassie stained gels of these samples confirmed this notion as the level of a 55‐kDa protein, corresponding to Rubisco, was strongly depleted in nuclear protein samples compared with the total leaf extract (Fig. 1b). An additional two biological replicates for which similar results were obtained were generated for which results are presented (Fig. S2).

### Quantitative label‐free LC‐MSMS allows the identification and quantification of the complex nuclear proteome

We sought to identify the changes in the host nuclear proteome associated with *P. capsici* infection. Protein extracts from enriched nuclei were fractionated, digested and analysed by LC‐MSMS. maxquant (Cox & Mann, 2008) was used to both identify and quantify proteins. Searches against the tomato (ITAG 2.4) and *P. capsici* (Phyca11) proteomes identified a total of 3975 protein groups (including *P. capsici* proteins) across our experiment (infected and noninfected samples, two time‐points and three biological replicates) (Notes S4).

**Figure 2:**
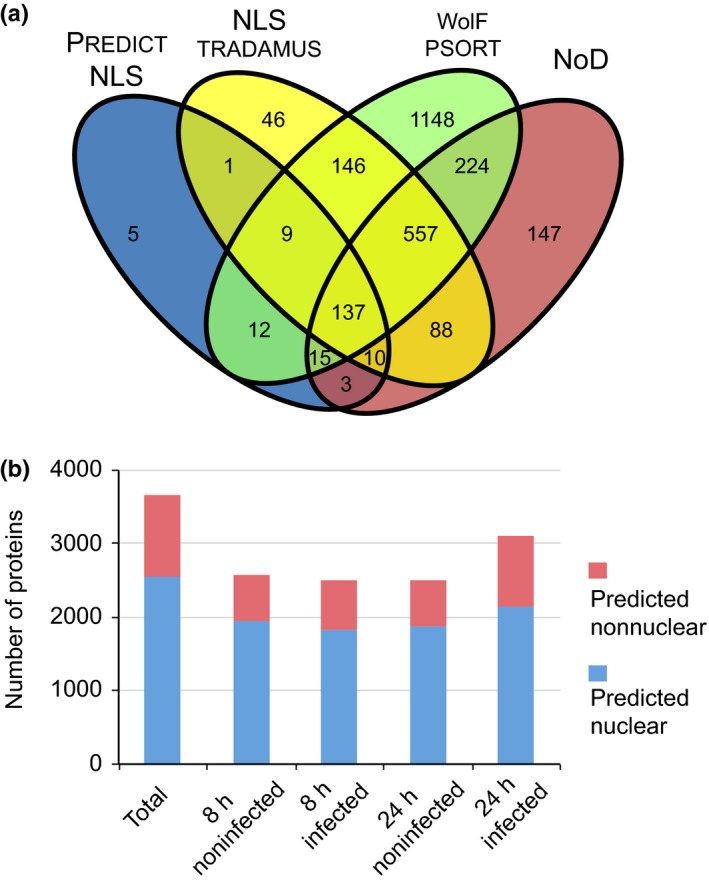
Amino acid sequence analysis reveals that the majority of identified proteins have a predicted nuclear association. (a) Contaminant proteins and *Phytophthora capsici* proteins were removed from the data set and all remaining proteins were subjected to four nuclear prediction tools to establish how many proteins had a predicted nuclear association. In total, 2548 proteins were either found to have a nuclear localization signal (NLS) (according to predictnls and nlstradamus) or a nucleolar localization signal (using nod) or were predicted to localize to the nucleus according to wolf p‐sort. (b) Overview of protein numbers found within individual treatments and their predicted localization. Proteins that were not predicted to be nuclear in our analyses are classified as ‘predicted nonnuclear’. The full list of proteins found within the data set along with their quantification values is provided in Supporting Information Notes S1.

Before quantitative analyses of the tomato proteome, we assessed levels of nuclear enrichment while simultaneously defining nuclear proteome composition. For this, we removed proteins predicted to originate from *P. capsici*. This yielded a set of 3654 candidate nuclear protein groups. To gain further confidence in our data set, we predicted the localization of our candidate proteins. Out of our set of 3654 candidates, 2548 (69.7%) tomato proteins either carried a predictable nuclear localization signal or a nucleolar localization signal or were predicted to localize to the nucleus according to wolf psort (Fig. 2a). For 1106 proteins, computational predictions failed to explain their detection in our nuclear enriched samples, suggesting high false negative prediction rates, alternative nuclear import mechanisms or cytosolic contamination. We assessed overall protein detection rates as determined by the number of proteins identified and calculated the percentage of nuclear proteins found in each treatment sample (Fig. 2b). These analyses revealed similar protein detection rates and, as expected, little variation in the proportion of predicted nuclear proteins (75.5%, 73.1% and 75% for 8‐h noninfected, 8‐h infected and 24‐h noninfected samples, respectively) (Fig. 2b). Slightly more proteins (3114) were identified in our nuclear enriched samples 24 h post‐infection, where 68.9% were predicted to be nuclear (Fig. 2b). These results suggest successful enrichment of host nuclei in our experiments and subsequent reliable detection of nuclear proteins across all our treatments. From these observations, we surmised that quantitation of dynamic changes should be feasible.

### *Phytophthora capsici* infection results in drastic and consistent changes in the host nuclear proteome

**Figure 3:**
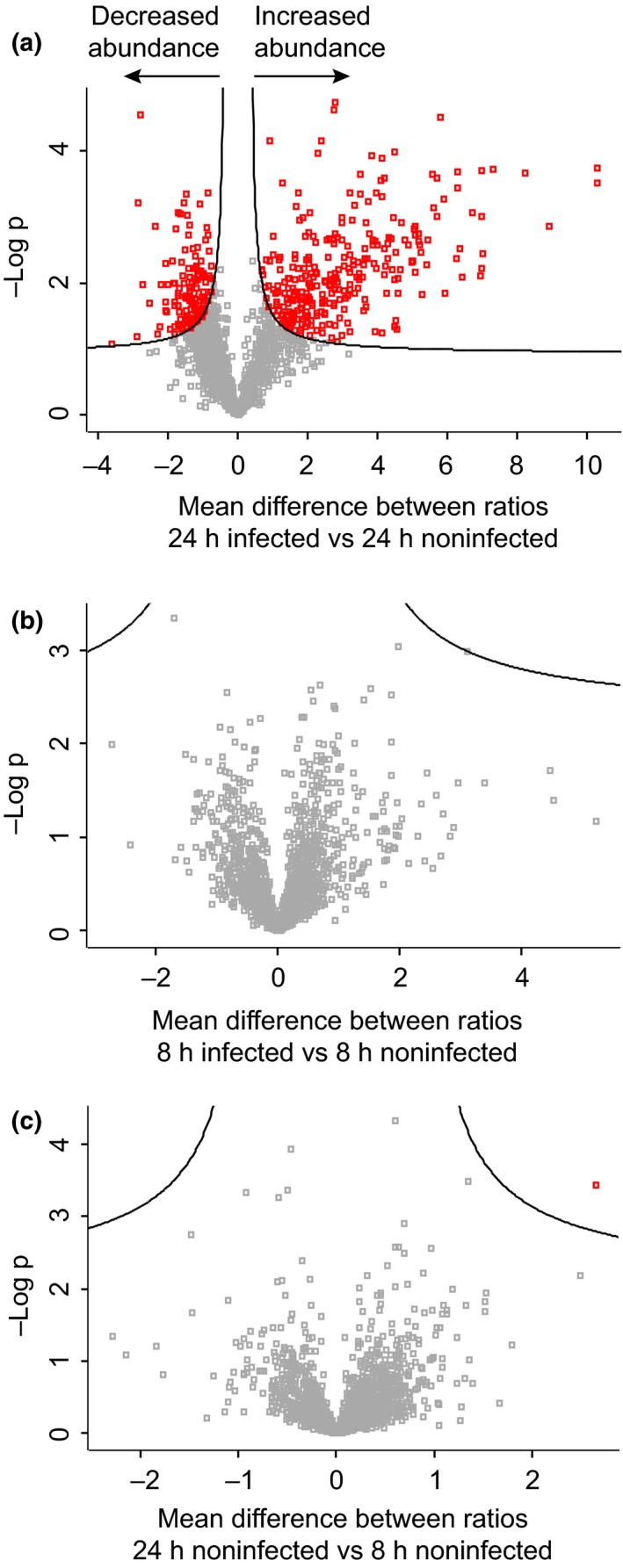
Identification of proteins that show a significant change in abundance during infection. Protein expression data were analysed within perseus using two‐sample *t*‐tests and a false discovery threshold of 0.05 to identify nuclear candidates significantly changing in abundance. A total of 285 proteins significantly increased in abundance 24 h post‐infection relative to the 24‐h noninfected sample, while 140 proteins significantly decreased in abundance upon infection (a). No proteins were found to be significantly altered in abundance 8 h post‐infection (b), while only one protein passed the significance threshold when comparing 8‐h and 24‐h noninfected samples (c).

To detect possible changes in the host nuclear proteome attributable to infection, we performed label‐free quantification (LFQ) analysis on all tomato proteins identified within our data set (Fig. S1). LFQ values were normalized to adjust for any differences introduced as a result of sample handling or fractionation using the delayed normalization approach in maxquant (Cox *et al*., 2014). Correlation plots for LFQ intensities revealed strong correlation between biological replicates within treatments, suggesting successful sample loading and normalization (Fig. S1). All proteins for which LFQ data was available in both infected and noninfected samples, and across all three biological replicates, were analysed within perseus to identify significant changes in protein abundance (data summarized in Fig. 3, Table 1 and Notes S5). These analyses identified 285 proteins with increased abundance and 140 proteins with reduced levels in infected samples, 24 h after infection (Fig. 3; Table 1). Comparison of the 24‐ and 8‐h infected samples also showed significant changes in protein abundance (Table 1). Comparisons of protein lists revealed considerable overlap, suggesting relatively minor changes to the nuclear proteome in the early stages of infection (Fig. S4). Consistent with this view, our perseus analyses failed to identify any proteins with significantly altered abundance between the 8‐h infected and noninfected treatments, suggestive of subtle changes in the nuclear proteome that are masked by variation between replicates (Table 1; Fig. 3b). Importantly, only one protein was significantly altered in abundance when comparing the noninfected samples at 8 and 24 h (Table 1; Fig. 3c), providing a high degree of confidence in sample treatment, handling and subsequent protein quantification.

**Table 1:** Protein quantification data

Treatment	Number of proteins significantly changing in abundance		Number of proteins with consistent presence/absence expression profile	
	Increased abundance	Decreased abundance	Increased abundance	Decreased abundance
8‐h I vs 8‐h NI	0	0	14	18
24‐h I vs 24‐h NI	285	140	222	33
24‐h I vs 8‐h I	240	88	163	10
24‐h NI vs 8‐h NI	1	0	11	5

All proteins for which label‐free quantification (LFQ) data were available in both infected and noninfected samples and across all three biological replicates were analysed within perseus to identify significant changes in protein expression. Where LFQ values were missing, the data were filtered to identify those proteins with a consistent presence/absence expression pattern. Increased abundance indicates those proteins showing greater abundance in treatment A vs treatment B, while decreased abundance indicates those proteins showing reduced abundance in treatment A vs treatment B. I, infected samples; NI, noninfected samples.

A considerable number of proteins had missing LFQ values in one or more samples. Missing LFQ values can result from absence or low levels of a protein within a sample, leading to peptide intensity values that fall below the LFQ intensity threshold. We surmised that infection‐associated changes in protein levels could lead to *P. capsici*‐specific gains or losses of LFQ values in our experiments. We therefore opted to select proteins that feature a consistent presence/absence profile across all our replicates and define those as differentially expressed (data summarized in Table 1 and Notes S6). These analyses yielded 32 differentially abundant proteins in infected vs noninfected comparisons at the 8‐h time‐point and 255 candidates differentially present at 24 h (infected vs noninfected). A summary describing these proteins is provided in Notes S7.

### Proteins implicated in diverse host processes show changes in abundance during *P. capsici* infection

**Figure 4:**
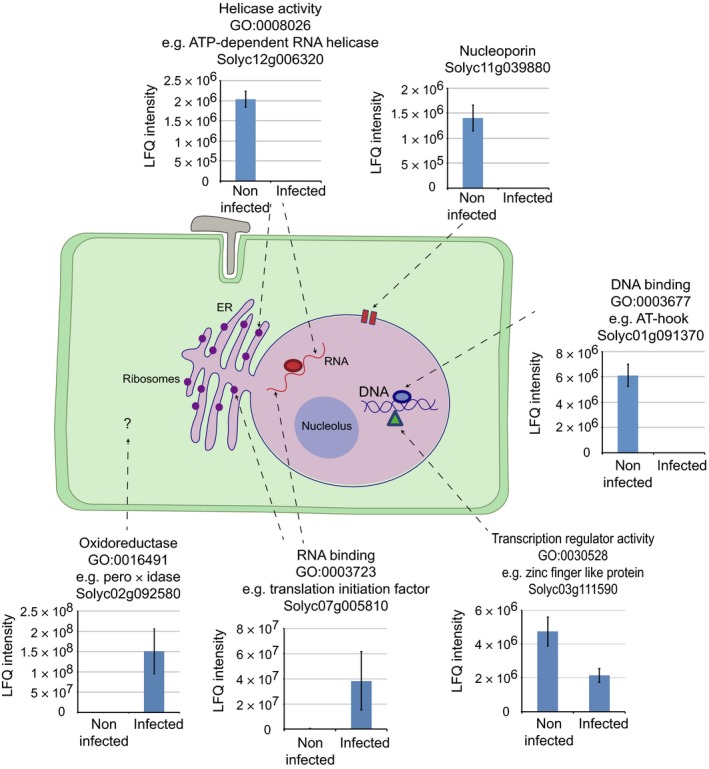
Overview of host cellular processes changing during *Phytophthora capsici* infection. Quantitative proteomics revealed that diverse nuclear and nuclear‐associated processes may be modified during pathogen infection, including the activity of DNA‐ and RNA‐binding proteins, transcription factors, components of the nuclear pore complex and DNA and RNA helicases. Predicted nonnuclear proteins including peroxidases were also found to change in abundance. Graphs show the label‐free quantification data (LFQ) for selected proteins, with each bar representing the average LFQ intensity from three biological replicates ± SD.

Given the observation that large numbers of proteins were found to change in abundance 24 h post infection relative to noninfected samples (507 with increased and 173 with decreased abundance), we examined the predicted functions of these proteins, to identify host processes that may be perturbed during infection. Using proteins with available gene ontologies (GOs), we ranked terms according to frequency for those proteins that showed increased or decreased abundance 24 h post‐infection (Fig. S5). GO terms associated with protein binding (GO:0005515), nucleic acid binding (GO:0003676), nucleotide binding (GO:0000166), hydrolase activity (GO:0016787) and transferase activity (GO:0016740) were highly ranked in proteins that both increase and decrease in abundance during infection (Fig. S5). In addition, proteins annotated with oxidoreductase activity (GO:0016491) and ribonucleotide binding activity (GO:0032553) were highly ranked in proteins which increased in abundance during infection, while predicted helicase activity (GO:0008026), RNA binding (GO:0003723) and transcription regulator activity (GO:0030528) were among the top GO terms for those proteins that decreased in abundance during infection (Fig. S5). A selection of proteins with these common GO terms along with their abundance profile and localization pattern is presented in Fig. 4. It should be noted, however, that none of these terms were significantly enriched against control background sets.

### Levels of AT‐Hook‐Like proteins dynamically change during infection

We identified many host proteins that dynamically change in abundance as a consequence of *P. capsici* infection. These include regulators of immunity characterized in other plant species as well as a large number of proteins with hitherto undefined roles in immunity. To learn more about immunity‐associated processes in plants, we decided to focus on the AHL protein family, a relatively uncharacterized set of proteins found in plants. We interrogated our full data set and identified 16 AHL proteins that were detected in our study and represent all AHL types known in plants (Table 2; Notes S8). Interestingly and despite representation of all AHL types in our data set, only type II and III members were found to be differentially abundant (depleted in infected tissues) in our experiments, suggesting a specific role in immunity or susceptibility to *P. capsici*.

**Table 2:** AT‐Hook‐Like (AHL) proteins selected for follow‐up experiments

Protein identifier	AHL number	AT‐hook motif type	PPC type	AHL type	Protein abundance
Solyc01g091370	AHL1	II	B	III	Not detected 24 h PI
Solyc08g008030	AHL5	II	B		Decreased abundance 24 h PI (2‐fold down)
Solyc01g094460	AHL9	I + II	B	II	Decreased abundance 24 h PI (2.8‐fold down)
Solyc04g076220	AHL17a	I	A	I	No significant change 24 h PI
Solyc12g087950	AHL17b	I	A		No significant change 24 h PI

PI, post infection; PPC, Plants and Prokaryotes Conserved.

### AHL proteins localize to the nucleus

**Figure 5:**
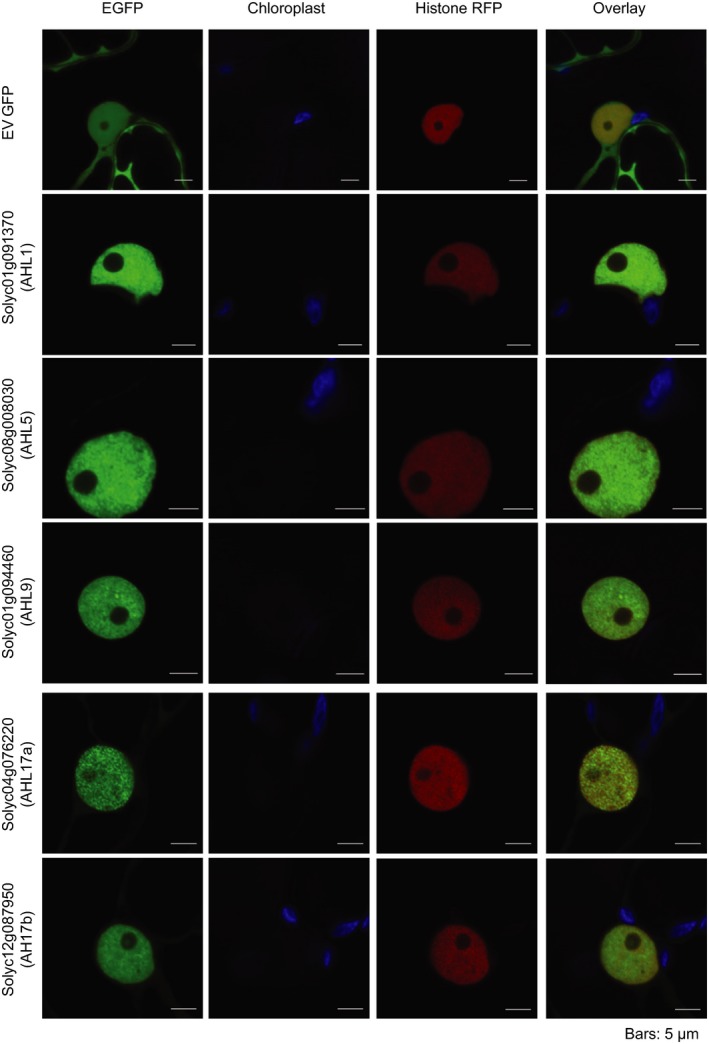
Localization of AT‐Hook‐Like (AHL) proteins *in planta*. AHL1, AHL5, AHL9, AHL17a and AHL17b localized to the plant nucleus when transiently overexpressed in *Nicotiana benthamiana* using *Agrobacterium tumefaciens*‐mediated expression. *Nicotiana benthamiana* plants expressing histone‐RFP were used to visualize the nucleus. Leaves were infiltrated at an optical density (OD_600_) of 0.1 and imaged after 48 h. Bars, 5 μM. EGFP, enhanced green fluorescent protein.

To comprehensively assess the significance of our observations and investigate the role of AHL proteins, we selected five family members representing each AHL type (Table 2). Of these, three were found to decrease in abundance 24 h post‐infection (types II and III: AHL1, AHL5 and AHL9) while two did not change in abundance during infection (type I: AHL17a and AHL17b) (Table 2). In order to determine the localization pattern of our five AHL proteins, EGFP fusion constructs were generated and resultant proteins expressed in *N. benthamiana* using *A. tumefaciens*‐mediated transient expression. Ectopic expression and subsequent western blot analyses revealed that all EGFP‐fusion proteins were detectable and, to some extent, susceptible to proteolytic cleavage of the EGFP epitope tag (Fig. S6). Despite this observation, localization of EGFP‐AHL proteins in transgenic *N. benthamiana* plants expressing RFP‐labelled histone 2B (Martin *et al*., 2009) revealed specific accumulation of all fusion proteins in the nucleus evidenced by co‐localization with the nuclear marker and low levels of fluorescence emanating from the cytosol (Fig. 5). These results suggest that all AHL proteins localize to the nucleus *in vivo*. These results are consistent with their detection in our proteomic analyses, thereby further validating our approach and results.

### Type II and III AHL proteins have roles in immunity to *P. capsici*

**Figure 6:**
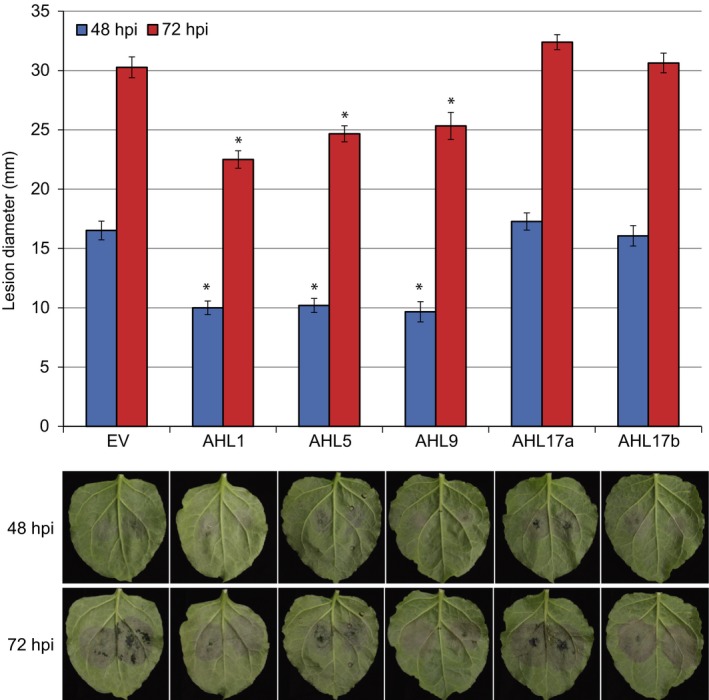
Impact of five tomato AT‐Hook‐Like (AHL) proteins on immunity to *Phytophthora capsici*. Over‐expression of AHL1, AHL5 and AHL9 resulted in reduced *P. capsici* growth compared with an empty vector (EV) control. Virulence assays were performed in *Nicotiana benthamiana* plants over‐expressing enhanced green fluorescent protein (EGFP)‐AHLs. Plants were infiltrated with the *Agrobacterium tumefaciens* expression construct at OD 0.1. After 48 h, leaves were detached and 10‐μl drops of *P. capsici* spores (at a density of 50 000 ml^−1^) were placed on the leaf surface. Lesion diameters were measured 48 and 72 h post‐infection (hpi). Significant difference relative to the EV control according to Dunnett's multiple comparison testing: *, *P *<* *0.05. Error bars show ± SE.

Three AHL proteins showed lower abundance levels upon *P. capsici* infection. We hypothesized that *P. capsici* targets specific host nuclear processes that affect AHL protein levels, which leads to suppression of immunity. If this is the case, over‐expression of AHL proteins may circumvent these events and impair *P. capsici* infection. Over‐expression of three AHL proteins (AHL1, AHL5 and AHL9) consistently reduced *P. capsici* lesion growth compared with the control (EGFP), whereas over‐expression of AHL17a and AHL17b had no significant impact on *P. capsici* lesion growth rates (Fig. 6). Interestingly, AHL over‐expression phenotypes were correlated with their behaviour in our data set (reduced abundance during *P. capsici* infection) and consistent with their recent classification, as the two type I AHLs (AHL17a and AHLb) had no effect on *P. capsici* growth, while all type II and III AHL proteins (AHL1, AHL5 and AHL9) caused a reduction in *P. capsici* growth upon over‐expression. These results suggest that (1) plants become more susceptible as a consequence of AHL protein level reduction and (2) some but not all AHL proteins are involved in immunity‐associated processes. Given the low degrees of similarity between the AHL proteins enhancing immunity (Fig. S7) and the overall diversity within this protein family, our data sets represents a valuable means to identify factors that may contribute to immunity.

**Figure 7:**
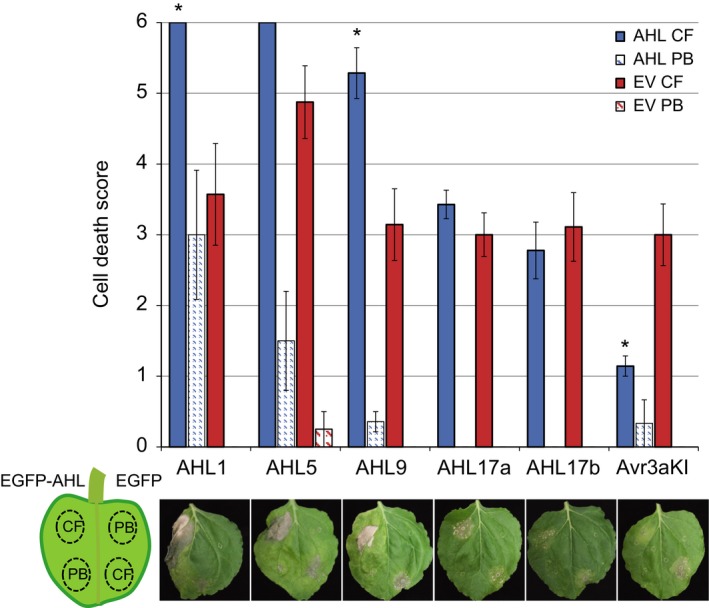
Two AT‐Hook‐Like (AHL) proteins that enhance immunity to *Phytophthora capsici* also impact upon pattern triggered immunity (PTI) responses. PTI assays were performed in *Nicotiana benthamiana* plants over‐expressing enhanced green fluorescent protein (EGFP)‐AHLs or EGFP‐Avr3a^KI^ (Engelhardt *et al*., 2012) on one half of the leaf and EGFP‐EV on the other. Leaves were then infiltrated with either *Phytophthora capsici* culture filtrate (CF) to trigger a PTI response, or pea broth (PB) as a negative control. Results show the levels of cell death observed 72 h after treatment with CF or PB. Cell death was scored from 0 to 6 (no cell death to high cell death) following the scoring method described by Stam *et al*. (2013). Significant difference relative to EV CF control according to Welch's *t*‐test: *P < 0.05. Error bars show ± SE. EV, empty vector.

Given that ectopic expression of AHL protein affects immunity to *P. capsici*, we asked whether this phenotype is attributable to enhanced PTI responses. To test this, we expressed EGFP‐AHL fusion proteins along with EGFP alone in *N. benthamiana* leaves, after which panels were treated with *P. capsici* CF and PB as a negative control (Fig. 7). The *Phytophthora infestans* RXLR effector AVR3a^KI^, a known suppressor of PTI cell death, was used as a positive control (Bos *et al*., 2010). We found that, upon expression of AHL1 and AHL9, PTI responses elicited by CF treatment were significantly enhanced compared with control leaf panels (Fig. 7). Although AHL5 expression led to levels of cell death similar to those produced by AHL1 and AHL9 expression, AHL5 expression may have enhanced the response of empty vector (EV) infiltrated panels, suggesting systemic effects in this assay and thereby rendering the comparisons nonsignificant. Expression of EGFP‐AVR3a^KI^ led to suppression of cell death, indicating that CF treatment induced PTI responses (Fig. 7). Finally, expression of AH17a and AHL17b did not have a significant impact on cell death, indicating roles in host processes not involved in PTI (Fig. 7). These results show that AHL1, AHL9 and possibly AHL5 enhance PTI response pathways and immunity to *P. capsici*.

## Discussion

Despite available plant genome sequences and gene expression data across various developmental stages or stresses, little information is available about plant cell proteomes and nuclear protein repertoires in particular. This is particularly evident in crops, where only modest progress has been made in understanding the processes that underpin complex traits such as pathogen resistance. While transcriptome‐based studies provide valuable information on mRNA abundance across treatments, quantitative proteomics can greatly complement such data sets as only a fraction of mRNAs are translated and the stability of their products varies depending on a plethora of possible post‐translational modifications and mechanisms. Studies on proteomes are therefore pivotal, if the biology of immunity is to be fully understood.

To understand the interaction between tomato and *P. capsici* and reveal which proteins may act as immunity factors in crops, we identified and quantified nuclear proteins from tomato whose abundance changed during infection, using organelle enrichment and quantitative proteomics. Analyses of our nuclear enriched protein samples revealed drastic depletion of cytosolic and some retention of ER resident proteins, respectively (Fig. 1), suggesting that nuclear enrichment is not absolute. Given that the ER forms a contiguous network with the nuclear envelope (Hetzer, 2010), we would expect ER resident proteins to be found within our nuclear enriched samples, and this should be considered when interpreting our results.

We applied nuclear prediction algorithms to identify those proteins for which there is evidence of nuclear targeting. This revealed that the majority of identified proteins (2548) had a predicted nuclear association while 1106 proteins did not (Fig. 2). The significant differences in gene lists emanating from these prediction algorithms indicate varying levels of performance and suggest the presence of false negatives in our data set (i.e. a protein detected in our study that is predicted to be nonnuclear). In addition, proteins for which nuclear localization has not been inferred may predominantly reside outwith the nucleus but shuttle during infection. In either scenario, our data form a valuable resource to mine for proteins that reside in the plant nucleus either permanently or upon specific (biotic) cues.

Quantitative analyses revealed evidence of significant changes in the tomato nuclear proteome and will serve as a valuable experimental database suited to describe the cellular events underpinning *P. capsici*−tomato interactions. Hundreds of proteins were found to consistently change in abundance 24 h post infection. Proteins found to decrease in abundance include a number of predicted DNA‐binding proteins, transcription factors and LRR serine/threonine kinases, while those proteins found to increase in abundance during infection include a number of predicted RNA‐binding proteins and proteins implicated in metabolic processes and translation, suggesting that diverse nuclear processes are affected by infection. Interestingly, very few proteins were found to significantly change in abundance 8 h post‐infection. At the early stages of infection there is likely to be a relatively low proportion of infected cells and only small differences in protein abundance, meaning that few proteins will pass robust and stringent statistical analyses. This suggests that the detection of false positive changes in abundance is likely to be very low. It also means that, if one wishes to identify subtle changes at early infection time‐points, the number of replicates used should probably be increased.

Among the proteins that changed in abundance, we identified a significant number of defence‐associated proteins. These include transcription factors (TFs) such as a WRKY‐like TF (Solyc09g014990.2.1), which increased in abundance during infection, and a helix‐loop‐helix TF (Solyc01g096370.2.1) whose levels dropped during infection. The WRKY‐like TF is homologous to WRKY33 from *Arabidopsis thaliana,* which is a key defence‐associated transcriptional regulator (Zheng *et al*., 2006), while helix‐loop‐helix TFs have been shown to regulate plant defence (Song *et al*., 2013; Woldemariam *et al*., 2013). In addition, RNA helicases, proteins that regulate RNA structure, metabolism and gene expression (Li *et al*., 2008), were found to decrease in abundance during infection. Given that silencing of a putative RNA helicase in *A. thaliana* and *N. benthamiana* was shown to cause enhanced susceptibility to *Phytophthora* (Qiao *et al*., 2015), it is plausible that *P. capsici* targets RNA helicases to modify host RNA metabolism to suppress immunity. We also observed significant drops in abundance for three nucleoporins (Solyc04g007280.2.1, Solyc01g102540.2.1 and Solyc11g039880.1.1) during infection. Nucleoporins help form the plant nucleopore complex, which regulates protein trafficking across the nuclear envelope. In *A. thaliana*, mutating nucleoporin genes negatively impacts immunity (Zhang & Li, 2005; Wiermer *et al*., 2010). Given the important role nucleocytoplasmic trafficking plays in plant defence (Wiermer *et al*., 2007), an intriguing possibility is that *P. capsici* perturbs the transport of defence regulators or signalling proteins into and out of the nucleus to inhibit host defence. Our data set thus forms a platform from which factors that require nucleoporin can be identified.

Our results suggest that regulators of immunity are frequently changing in abundance during virulent interactions, which, by extension, implies that other proteins with unknown function but with similar abundance profiles contribute to immunity‐associated processes. We tested this notion by selecting and testing a set of nuclear proteins whose abundance changed during infection. This led us to identify and investigate five members of the AHL protein family, all of which were detected in our study.

Three of these AHL proteins (AHL1, AHL5 and AHL9) decreased in abundance 24 h post‐infection, while levels of AHL17a and AHL17b appeared unchanged. Over‐expression of AHL1, AHL5 and AHL9 in *N. benthamiana* caused enhanced resistance to *P. capsici* (Fig. 6), whereas AHL17a and AHL17b had no visible impact on infection. Further functional analyses revealed AHL1 and AHL9 to be acting upon PTI response pathways, as their expression accelerated PTI‐associated cell death responses upon culture filtrate treatments in *N. benthamiana* (Fig. 7).

AHL protein family members have been previously implicated in immunity in pepper (*Capsicum annuum*) and *A. thaliana*. Over‐expression of the *C. annuum* CaATL1 gene in transgenic tomato plants led to a reduction in growth of *Pseudomonas syringae* pv. *tomato* DC3000 and *P. capsici* compared with empty vector control plants (Kim *et al*., 2007). Conversely, microarray analysis has shown that over‐expression of *A. thaliana* AHL27 (Zhao *et al*., 2013), suppresses jasmonic acid (JA), abscisic acid (ABA), salicylic acid (SA) and ethylene (ET) signalling pathways (Lim *et al*., 2007). Given the roles of these pathways in innate immunity (Pieterse *et al*., 2012), ORE7/ESC/AHL27 is considered a negative regulator of immune responses. A previous report also identified AHL20, AHL15, AHL19 and AHL27 as negative regulators of plant immunity in *A. thaliana*, evidenced by a reduction in FRK1 (flg22 (N‐terminal part of flagellin)‐induced receptor‐like kinase 1) expression after treatment of protoplasts, over‐expressing AHL proteins, with flg22 (a 22 amino acid peptide derived from bacterial flagellin) (Lu *et al*., 2010).

Our results provide an intriguing glance into the signalling pathways that lead to PTI and enhanced immunity. Although many regulators involved in perception, generation and transduction of PAMP‐triggered signalling have been identified, little is yet known about the factors that lie downstream and act in the nucleus. As AHL proteins are thought to bind DNA, studying their function may thus reveal processes required for the generation of a PTI output. We noted that AHL5 seemed to enhance immunity to *P. capsici*, while we did not observe any significant impact on PTI responses when it was over‐expressed in our assay (Fig. 7). In leaves where AHL5 was expressed, EV control panels had an enhanced PTI response compared with other control infiltrations (for AHL1 and AHL9) (Fig. 7). In addition, leaf panels over‐expressing AHL5 already showed low levels of cell death when treated with PB (negative control), possibly further masking an enhanced PTI response. Modified experimental set‐ups may help to elucidate the basis of our results and reveal a possible positive role for AHL5 in PTI regulation.

To clarify our results and add to our understanding of AHL protein function, we assessed AHL type membership as defined by Zhao *et al*. (2013), and revealed that type II and III AHLs tested in this study have positive roles in immunity in tomato. These results contrasted with observations made upon over‐expression of AHL17a and AHL17b, where no great differences in immunity were observed in our experiments. Further cloning and testing of additional AHL proteins from either type may help to answer the question of whether AHL classification is predictive of a role in immunity. AT‐hook‐containing proteins are known to target and bind AT‐rich DNA sequences via the minor groove of DNA. All AHL proteins feature an AT‐hook motif and a Plants and Prokaryotes Conserved (PPC) domain, which is also commonly annotated as Domain of Unknown Function 296 (DUF296) (Zhao *et al*., 2013, 2014). Although AHL proteins are considered ancient transcription factors, the exact molecular function will need to be elucidated in plants. A set of robust phenotypes associated with over‐expression should allow further and valuable characterization of this elusive family *in vivo*.

In summary, we have combined nuclear enrichment with quantitative label‐free proteomics to identify hundreds of proteins with altered abundance during *P. capsici* infection. Type II and III AHLs were implicated in our study as regulators that impact *P. capsici*−host interactions. As the five AHLs investigated here represent hundreds of nuclear proteins that change in abundance during infection, we suggest that our data will help to identify additional proteins implicated in immunity against important pathogens. Given the central role the nucleus plays in plant−microbe signalling, dissecting the immune signalling pathways that are compromised during *P. capsici* infection will prove valuable in our efforts to bolster immunity in crops. This data set thus forms an important and rich resource that will be of use in follow‐on studies.

## Author contributions

A.J.M.H., V.M.H., G.B.M., K.H. and T.M.M.M.A. performed experiments. A.J.M.H., S.t.H. and E.H. designed experiments. A.J.M.H., R.S. and E.H. analysed data. A.J.M.H., G.B.M. and E.H. wrote the paper.

## Supporting information

Please note: Wiley Blackwell are not responsible for the content or functionality of any Supporting Information supplied by the authors. Any queries (other than missing material) should be directed to the *New Phytologist* Central Office.

**Fig. S1** Mass spectrometry data analysis pipeline.
**Fig. S2** Successful enrichment of nuclear proteins demonstrated by western blotting with anti‐histone H3 antibody and subcellular markers.
**Fig. S3** Plots describing levels of correlation between samples for each treatment and replicate.
**Fig. S4** Assessment of changes in protein levels between treatments.
**Fig. S5** Gene ontology (GO) term analysis for those proteins changing in abundance 24 h post‐infection.
**Fig. S6** Verification of the expression and stability of EGFP fusion constructs used for confocal microscopy, *Phytophthora capsici* infection and PTI assays.
**Fig. S7** clustalw alignment of AHL1, AHL5 and AHL9 amino acid sequences.
**Table S1** Primers used for generating constructs with stop codon and for insertion into pENTR‐D‐Topo

**Notes S1** Amino acid sequences for combined *Solanum lycopersicum* and *Phytophthora capsici* genomes.

**Notes S2** maxquant search parameters.

**Notes S3** Methods for filtering mass spectrometry data for nuclear predictions and missing LFQ values.

**Notes S4** Protein identification and quantification data for all proteins found within all treatments and replicates (‘proteinGroups.txt’ file used for downstream filtering and quantification).

**Notes S5** Proteins with significantly higher or significantly lower abundance during infection with *Phytophthora capsici*.

**Notes S6** Proteins with consistent presence/absence LFQ expression patterns across all three biological replicates when comparing infected and noninfected samples.

**Notes S7** Combined protein expression data for the 8‐ and 24‐h time‐points.

**Notes S8** Summary of At‐Hook‐Like (AHL) proteins detected in this study.
