# Supplementary material for: Quantitative analysis of the tomato nuclear proteome during Phytophthora capsici infection unveils regulators of immunity
Source: New Phytol. 2017 Apr 10;215(1):309–22. doi: 10.1111/nph.14540 (PMC5637918; doi:10.1111/nph.14540)
Supplement: Supplementary file 1 — Fig. S1 Mass spectrometry data analysis pipeline. Fig. S2 Successful enrichment of nuclear proteins demonstrated by western blotting with anti‐histone H3 antibody and subcellular markers. Fig. S3 Plots describing levels of correlation between samples for each treatment and replicate. Fig. S4 Assessment of changes in protein levels between treatments. Fig. S5 Gene ontology (GO) term analysis for those proteins changing in abundance 24 h post‐infection. Fig. S6 Verification of the expression and stability of EGFP fusion constructs used for confocal microscopy, Phytophthora capsici infection and PTI assays. Fig. S7 clustalw alignment of AHL1, AHL5 and AHL9 amino acid sequences. Table S1 Primers used for generating constructs with stop codon and for insertion into pENTR‐D‐Topo [file NPH-215-309-s001.pdf]

New Phytologist **Supporting Information**

Article title: Quantitative analyses of the tomato nuclear pro*teome during Phytophthora capsici* infection unveils regulators of immunity

Authors: Andrew J.M. Howden, Remco Stam, Victor Martinez Heredia, Graham B. Motion, Sara ten Have, Kelly Hodge, Tiago M.M.M. Amaro and Edgar Huitema
Article acceptance date: 07 February 2017

The following Supporting Information is available for this article:

Table S1 Primers used for generating constructs with stop codon and for insertion into pENTR-D-Topo.

Fig. S1 Mass spectrometry data analysis pipeline.

Fig. S2 Successful enrichment of nuclear proteins demonstrated by western blotting with anti-histone H3 antibody and subcellular markers.

Fig. S3 Plots describing levels of correlation between samples for each treatment and replicate.

Fig. S4 Assessment of changes in protein levels between treatments.

Fig. S5 Gene Ontology (GO) term analysis for those proteins changing in abundance 24 hours post-infection.

Fig. S6 Verification of the expression and stability of EGFP fusion constructs used for confocal microscopy, *P. capsici* infection and PTI assays.

Fig. S7 ClustalW Alignment of AHL1, AHL5 and AHL9 amino acid sequences.

Table S1 Primers used for generating constructs with stop codon and for insertion into pENTR-D-Topo.

Gene	Primer Name	Sequence
Solyc01g091370.2.1 (AHL1)	Solyc01g091370-F	CACCATGGAGTCAAGGGAAGCTAT
	Solyc01g091370-R	TTACTCTCCTTGCAATGATA
Solyc08g008030.2.1 (AHL5)	Solyc08g008030-F	CACCATGGATGGAAGAGAAGGGATG
	Solyc08g008030-R	TCATCCACGTGTCAGGTCAATC
Solyc01g094460.2.1 (AHL9)	Solyc01g094460-F	CACCATGGATCGAAGGGAAGCTATG
	Solyc01g094460-R	TCATCCTCGCATTAAATCAATG
Solyc04g076220.2.1 (AHL17a)	Solyc04g076220-F	CACCATGAAAAGGGAGTATGTACT
	Solyc04g076220-R	TCAGTAAGGCGGTGGTTGTGG
Solyc12g087950.2.1 (AHL17b)	Solyc12g087950-F	CACCATGAAAGAAAAATATATAGA
	Solyc12g087950-R	TTAGTAAGGCAGTAGTTGTCT

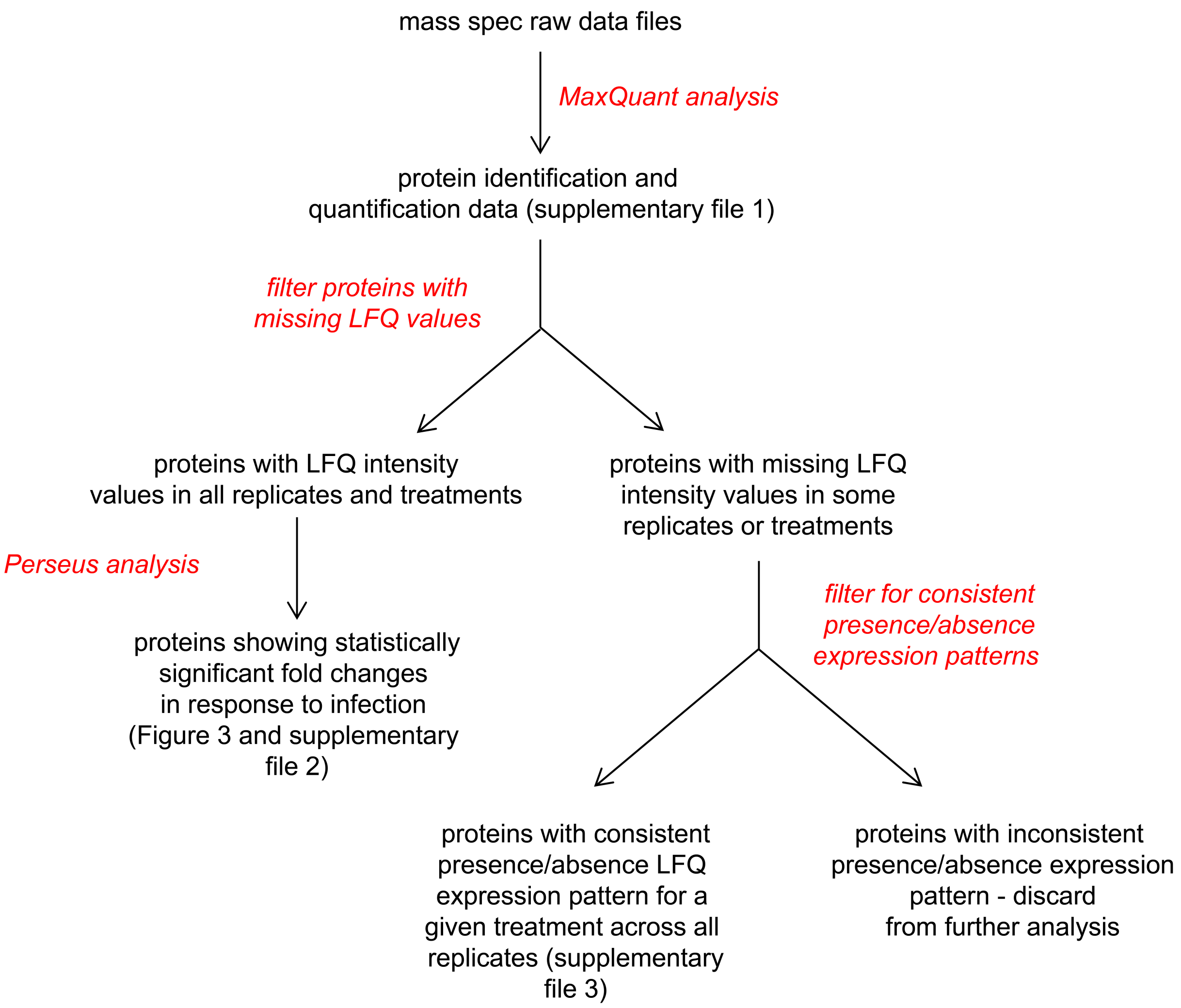

Fig. S1 Mass spectrometry data analysis pipeline.

Mass spectrometry data analysis pipeline. Mass spec results files were analysed using MaxQuant software to generate protein identification and label-free quantification data. Those proteins with label free quantification (LFQ) data available in all replicates and treatments were analysed within Perseus using two-sample t-tests and a false discovery threshold of 0.05 to identify those proteins showing significant changes in expression during infection. Those proteins with missing LFQ intensity values were analysed using the R software package to

identify consistent presence/absence expression patterns when comparing infected with non-infected samples.

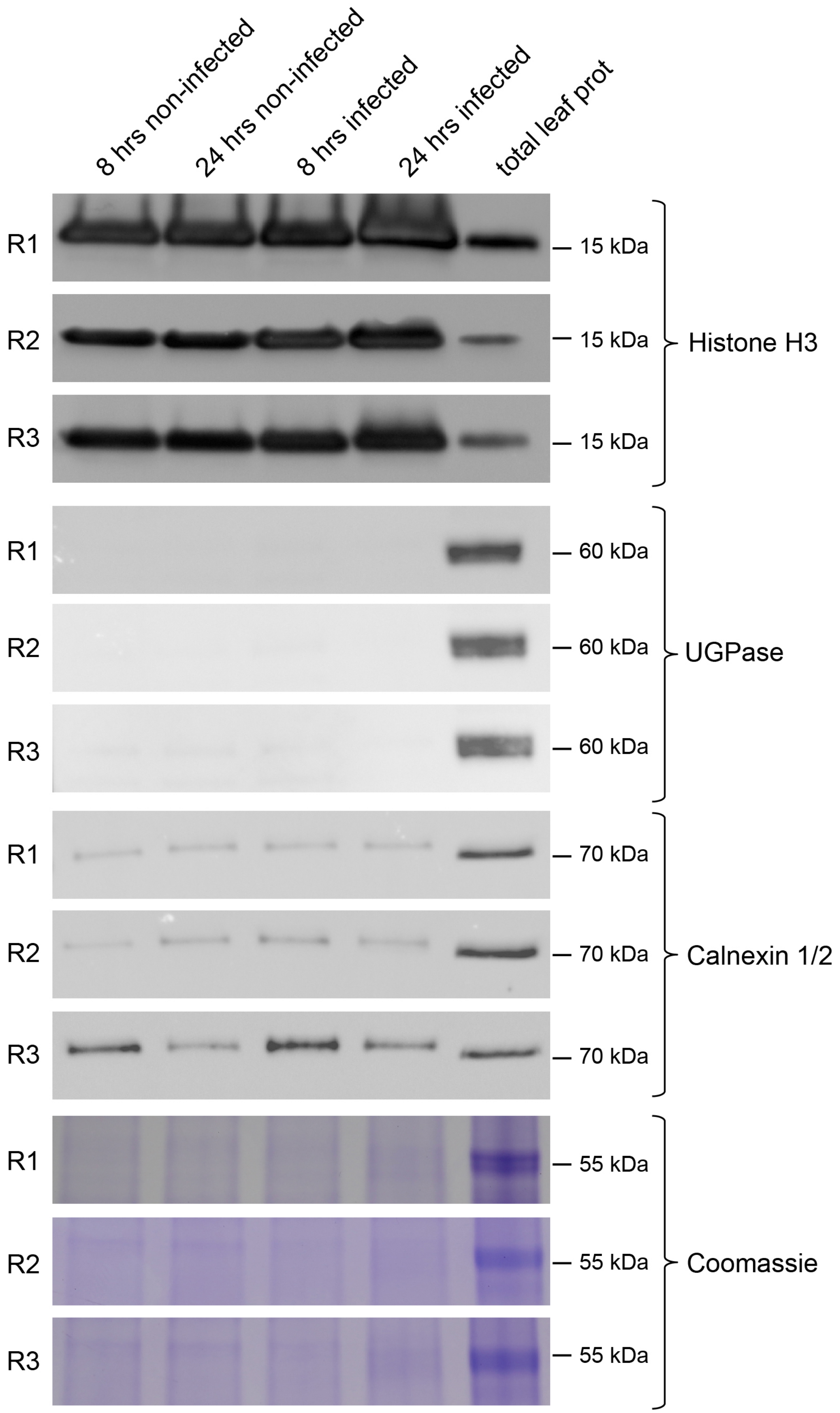
8 hrs non-infected
24 hrs non-infected
8 hrs infected
24 hrs infected
total leaf prot
R1
R2
R3
15 kDa
Histone H3
60 kDa
UGPase
70 kDa
Calnexin 1/2
55 kDa
Coomassie

Fig. S2 Successful enrichment of nuclear proteins demonstrated by western blotting with anti-histone H3 antibody and subcellular markers.

Protein extracts were generated from enriched nuclei samples and compared to a total protein extract from tomato leaves. Protein concentrations were adjusted for equal loading. Non-nuclear contamination was assessed by probing with anti-UDP-glucose pyrophosphorylase (UGPase) antibody (cytoplasm) and calnexin homolog 1/2 antibody (endoplasmic reticulum). Samples were also run on gels and coomassie stained to assess protein loading and RuBisCO abundance. The figure shows the results from 3 biological replicates (R1, R2 and R3) used for mass spectrometry experiments.

New Phytologist

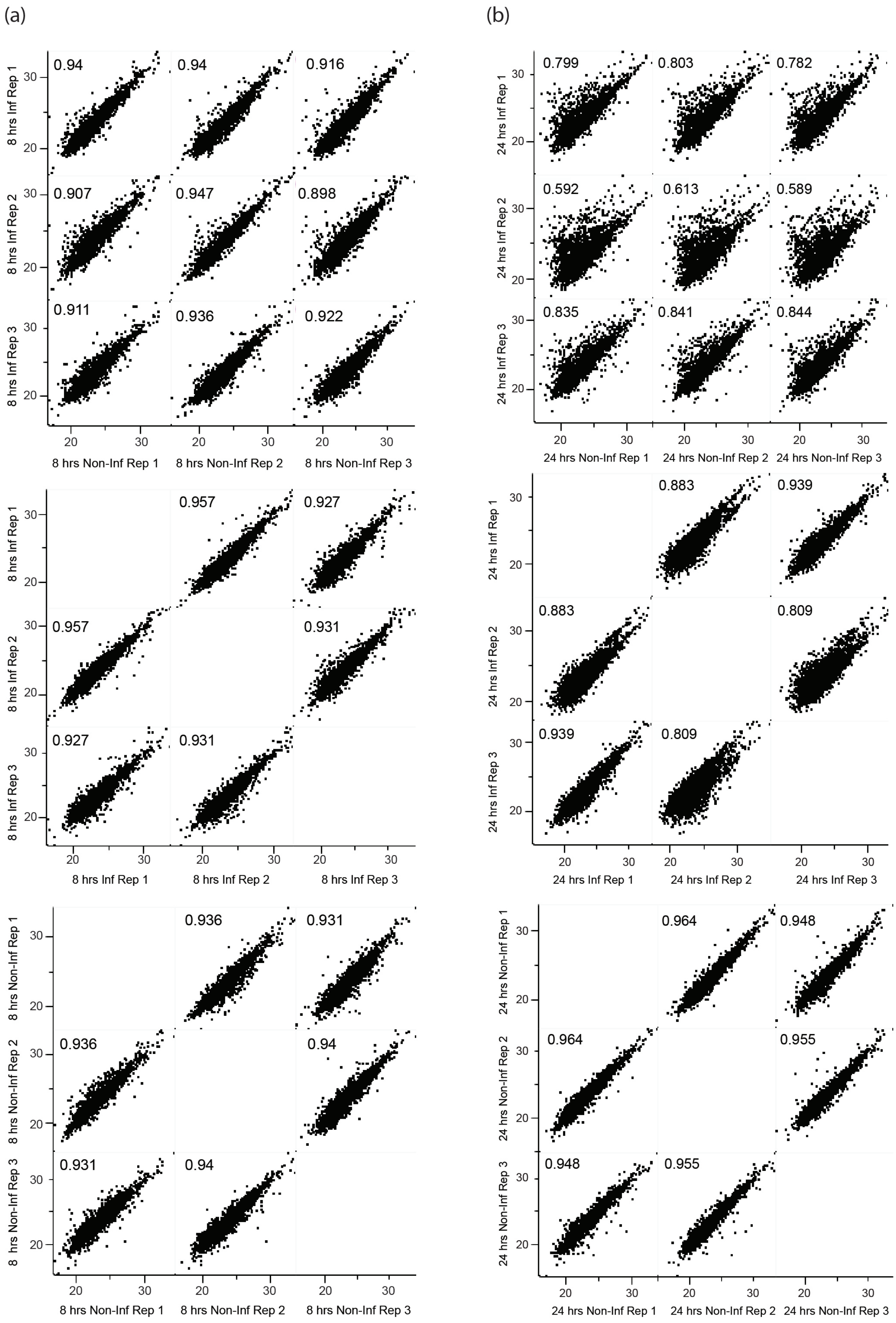

Fig. S3 Plots describing levels of correlation between samples for each treatment and replicate.

Pearson correlation values were calculated and are presented at the top left corner of each plot. **(a)** Correlation plots between infected and non-infected samples at the 8 hour time point. **(b)** Correlation plots between samples of infected and non-infected samples at the 24 hour time point.

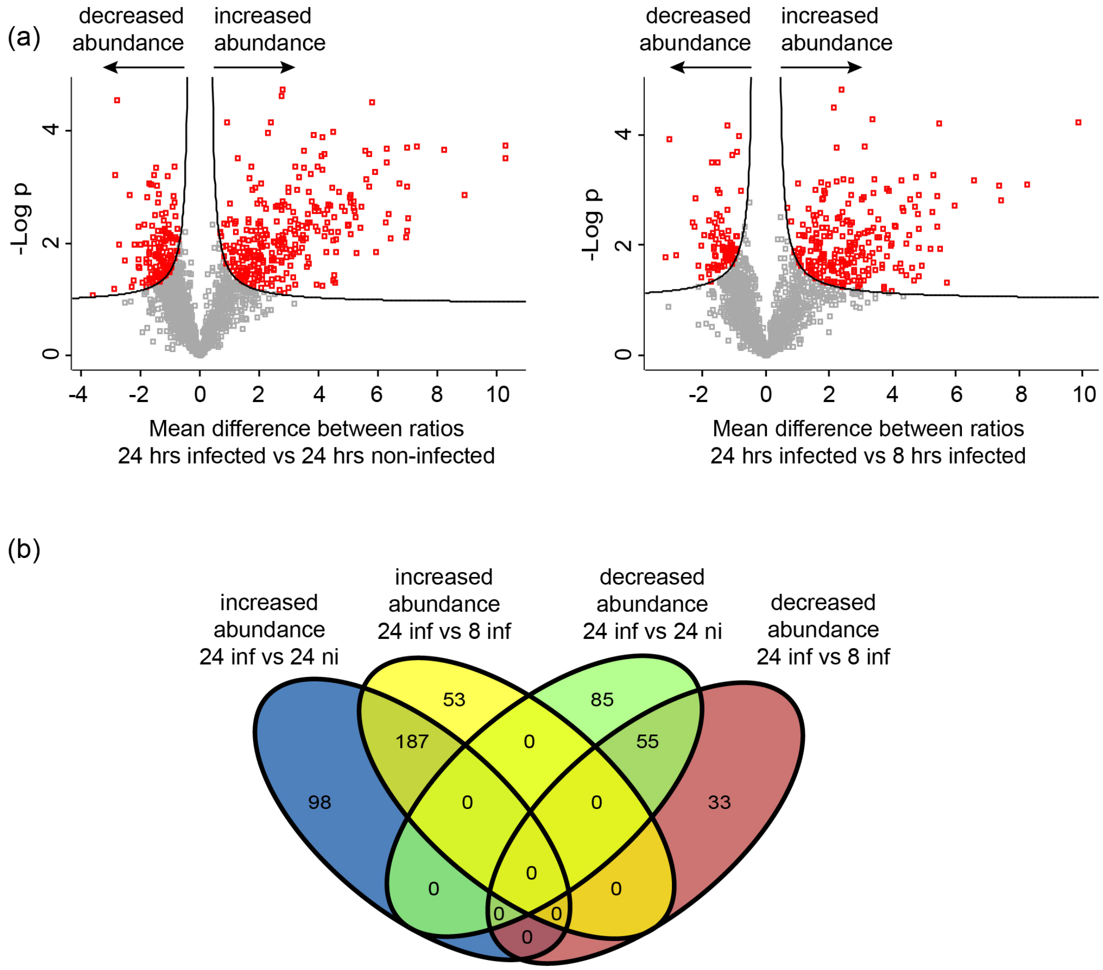

Fig. S4 Assessment of changes in protein levels between treatments.

(A) Protein expression scatter plots comparing 24 hrs infected with 24 hrs non-infected samples, and 24 hrs infected vs 8 hrs infected samples. **(B)** The venn diagram shows that many proteins changing in abundance 24 hrs post-infection vs 24 hrs non-infected also overlap with those proteins changing 24 hrs post-infection relative to 8 hrs post-infection.

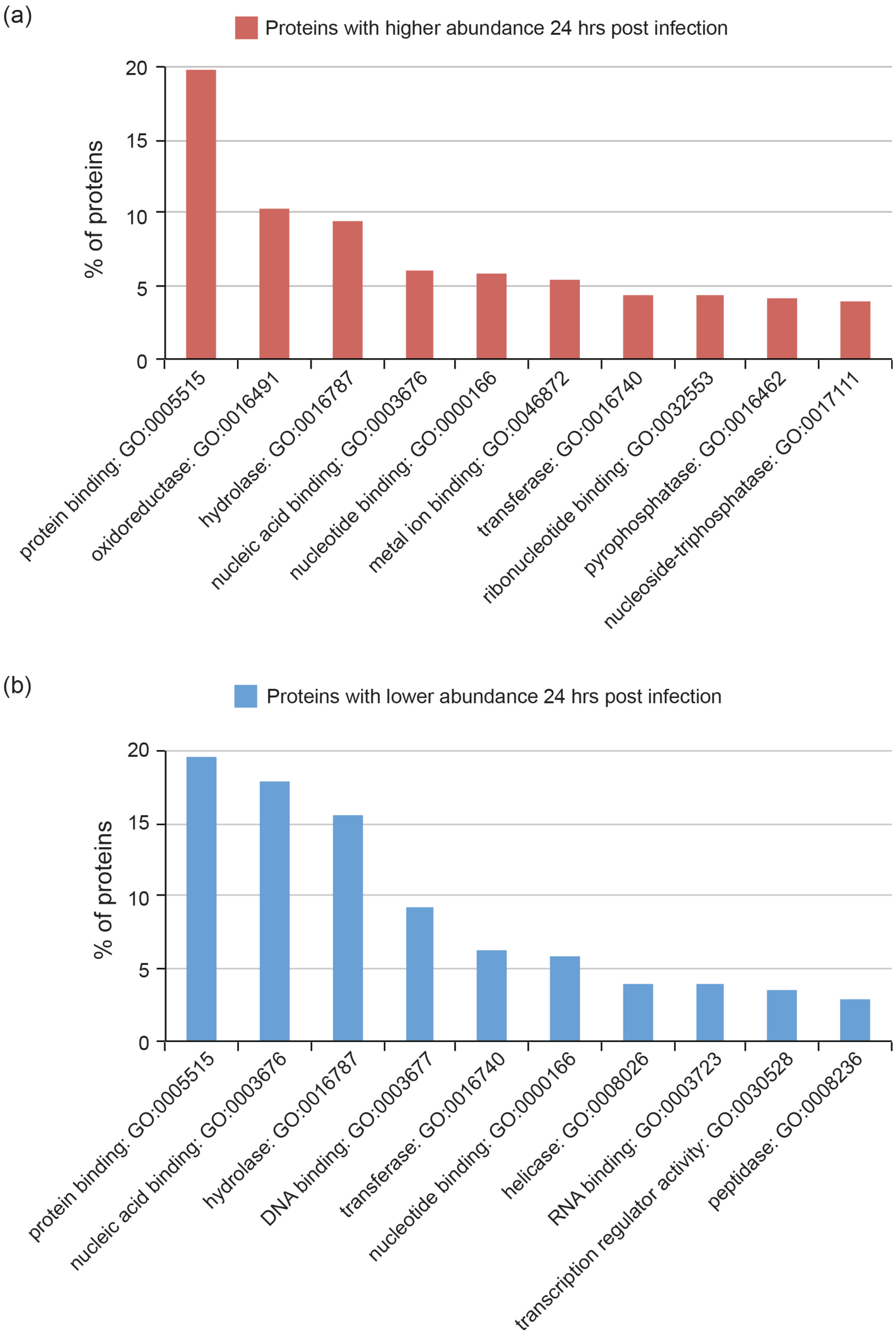
(a)
Proteins with higher abundance 24 hrs post infection
% of proteins
20
15
10
5
0
protein binding: GO:0005515
oxidoreductase: GO:0016491
hydrolase: GO:0016787
nucleic acid binding: GO:0003676
nucleotide binding: GO:0000166
metal ion binding: GO:0046872
transferase: GO:0016740
ribonucleotide binding: GO:0032553
pyrophosphatase: GO:0016462
nucleoside-triphosphatase: GO:0017111
(b)
Proteins with lower abundance 24 hrs post infection
% of proteins
20
15
10
5
0
protein binding: GO:0005515
nucleic acid binding: GO:0003676
hydrolase: GO:0016787
DNA binding: GO:0003677
transferase: GO:0016740
nucleotide binding: GO:0000166
helicase: GO:0008026
RNA binding: GO:0003723
transcription regulator activity: GO:0030528
peptidase: GO:0008236

Fig. S5 Gene Ontology (GO) term analysis for those proteins changing in abundance 24 hours post-infection.

The percentage of proteins with a particular GO term, relative to the total number of proteins changing in abundance, is provided for those proteins that show increased abundance **(a)** and those that show decreased abundance **(b)** 24 hours post-infection. The top 10 most common GO terms are presented for each category.

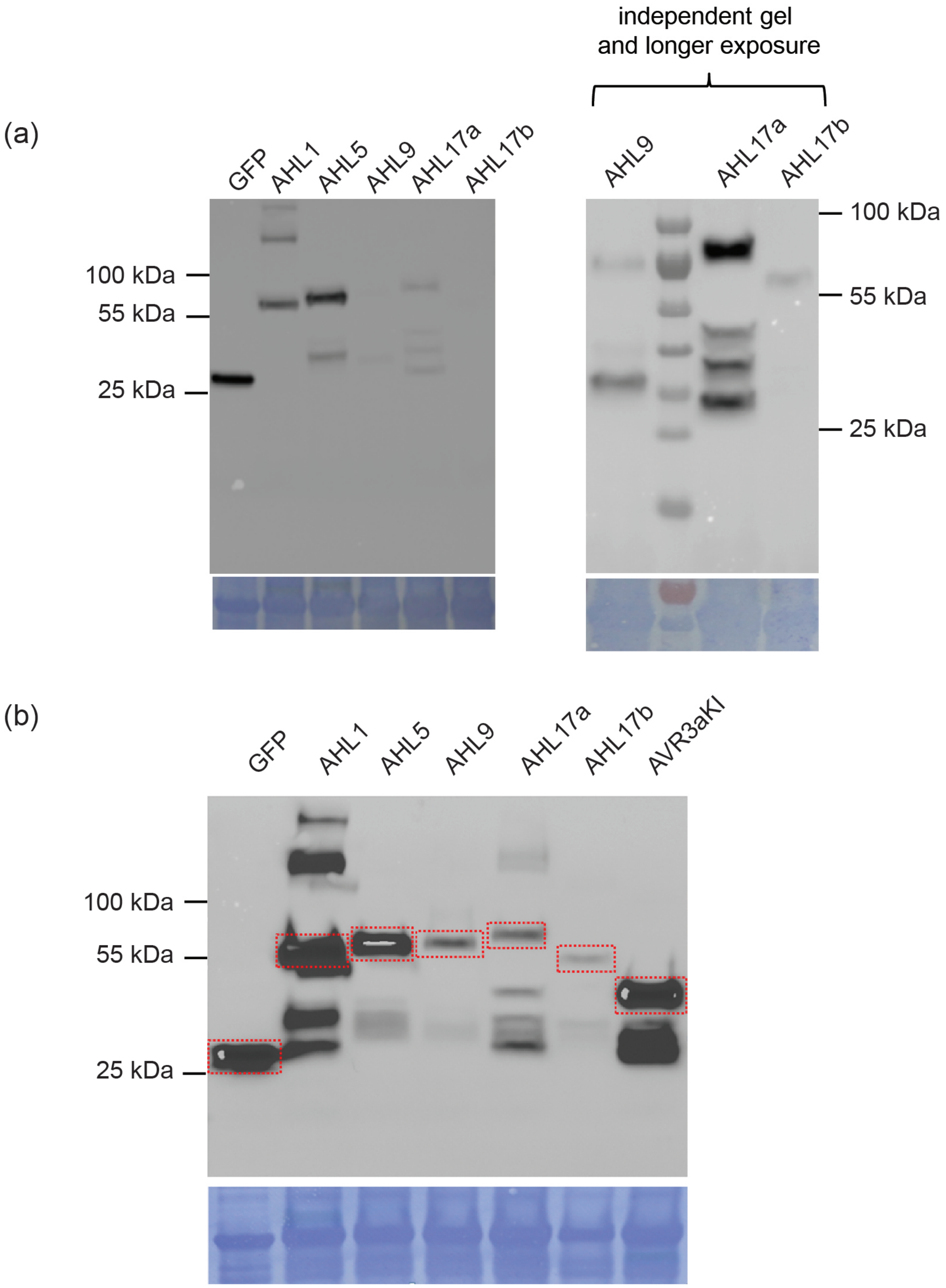

Fig. S6 Verification of the expression and stability of EGFP fusion constructs used for confocal microscopy, *P. capsici* infection and PTI assays.

A. tumefaciens AGL1 strains carrying EGFP-AHL fusions (vector pB7WGF2) were

infiltrated into *N. benthamiana* leaves at an OD600 of 0.1 (Panel A and B) or 0.05 (panel C). Samples were harvested 48 hours post-infiltration for protein extraction and western blotting with anti-EGFP antibody. **(A)** Western showing expression and stability of EGFP-AHL fusion proteins during localisation and *P. capsici* infection experiments. **(B)** Verification of EGFP-AHL protein expression and stability for PTI assays. Analyses of AHL9, AHL17a and AHL17b samples were repeated on a separate gel with longer exposure times to verify protein expression (right panel). **(C)** Detection of individual EGFP-AHL fusion proteins under conditions used for co-expression experiments (OD600 = 0.05). Loading was verified for each Western by means of Coomassie staining of each membrane.

```
CLUSTAL W (1.81) multiple sequence alignment

AHL1/1-318      MESREAIAPEAPSNYHM----------------VSRTESTPIPP-------------GT
AHL5/1-346      MDGREGMVLSGGAAYYLNRGISGSGSGVGGGSGAPTRVTTPPSYKSLSNPNISVQSNVGG
AHL9/1-339      MDRREAMMLPGSSPYYMQRGMSGSGSG-----NAPGLQGSPSINPSLTPNNIAFQSS-GS
                *: **.:   . : *::                      :..                *

AHL1/1-318      LAVIPPPV------------------ATAFPISSE--KKKRGRPRKYGPDGA-VARTISP
AHL5/1-346      GGTLSSTYQAVENPSPHFSHGINMSVVSSVSSGSDPVKKKRGRPRKYGPDGSKMSLGLSP
AHL9/1-339      GASIPQTL--VMDPSSTLSPRGSIGASSAMPQG-EPVRRKRGRPRKYGAQGA-MSLALTP
                 . :. .                    ::.. . :  ..*********.:*: ::  ::*

AHL1/1-318      ---TPISADFL---SKKVSVARPESEKKARNKVGAENLGEWISCSTGGNFLPHMITVEAG
AHL5/1-346      LSSTPSTGSIT--PGPKRAKGRPPGSGWKQK---LAPLGEWMNTSAGLAFTPHVINISVG
AHL9/1-339      ---PPSTQALSLNPTQKRGRGRPPGSGRKQQ---LTSFGGWLSNTAGIGFTPHVIMIAVG
                   .* :  :      * . .** .. . .:      :* *:. ::*  * **:* : .*

AHL1/1-318      EDVTMKIISFSQQGPRAICIISAVGLISNVTLRQPNSSGGTLTYEGRFEILSLSGSFTPT
AHL5/1-346      EDVAAKLLAFAEQRPRALCVLSASGSLSAVTLRPPTSSGSTVTYEGRFEILCLSGSYLVA
AHL9/1-339      EDITTKIMSFSQQGPRSICILSATGVISTVTLRQPSTSGGTVTYEGRFEILCLSGSFLVN
                **:: *:::*::* **::*::** * :* **** *.:**.*:*********.****:

AHL1/1-318      EFGGSRTTSRTGGMSISLASPDGRVVGGTLAGLLIAASPVQVVVGSFLPSNYQEVKPKKQ
AHL5/1-346      ESGGPR--DRTGGISISVSSPDGHVLGGAVGGRLIATSPVQVVVCSFV---Y-GPKVKSK
AHL9/1-339      ESGGSR--GRIGSLSVSLASPDGRVIGGGVGGVLVAASPIQVIVGSFL---CSSSKAKKR
                * **.*  .* *.:*:*::****.*:** :.* *:*:**:**:* **:       * *..

AHL1/1-318      KAELKAITYGTLSPAAPHSSNMEPRSSNAHTVNVPAAGTQNVISSSIQPNHWTAM-PSVQ
AHL5/1-346      ----------------PETSTKG---SEEESAEKPSIPIKATLSQDPTSNPATGVWPPIS
AHL9/1-339      ----------------AAESVQSAGTSDLQTTDNSVNPADALSNQNLAPSSSMGVWPSSR
                                .  *      *:  :.: .    .   ...  ..   .: *.

AHL1/1-318      DSRKSTTDINISLQGE
AHL5/1-346      RPELRNSQPEIDLTRG
AHL9/1-339      QIDLQTGHIDIDLMRG
                     .   :*.*
```

Fig. S7 ClustalW Alignment of AHL1, AHL5 and AHL9 amino acid sequences.

Identical or conserved residues are marked by “*” (asterisk) whereas “:” (colon) indicates strong conservation of amino acid properties between sequences. A “.” (period) indicates conservation on the basis of weakly similar amino acid properties.
