# Supplementary material for: Quantitative analysis of the tomato nuclear proteome during Phytophthora capsici infection unveils regulators of immunity
Source: New Phytol. 2017 Apr 10;215(1):309–22. doi: 10.1111/nph.14540 (PMC5637918; doi:10.1111/nph.14540)
Supplement: Supplementary file 4 — Notes S3 Methods for filtering mass spectrometry data for nuclear predictions and missing LFQ values. [file NPH-215-309-s004.rtf]

New Phytologist Supporting Information 

Article title: Quantitative analyses of the tomato nuclear proteome during Phytophthora capsici infection unveils regulators of immunity

Authors: Andrew J.M. Howden, Remco Stam, Victor Martinez Heredia, Graham B. Motion, Sara ten Have, Kelly Hodge, Tiago M.M.M. Amaro and Edgar Huitema

Article acceptance date: 22 February 2017 

Note S3 – Methods for filtering mass spec data for nuclear predictions and missing LFQ values.

The two Galaxy pipelines can be downloaded from https://github.com/remco-stam/nucprot/ 


## Pt 1. Data preprocessing

1. List with protein IDs manually extracted from Proteomics output file "proteinGroups.txt"
	- Shortened ID created by taking only gene number, not description
	e.g. from long IDs in column replace "\s" for "\t" and select first part, remove rest  
	- All Phyca11, "CON" and "REV" sequences were removed
	- Saved as Step1_proteomics_List.text

2. Edit supplied fasta file
	- Galaxy pipeline pt1 steps 1 to 4 show how the fasta file was: 
	transformed to table, descriptions removed, turned back into fasta
	- File saved as Step2_Tom_Pc.fasta
	- first entry was wrong, manually altered after downloading
	
3. Create fasta file with all sequences from proteomics data
	- Run create_subset.py
	specify List and Fasta file
	- Step3_Proteomics.fasta created, 3653 sequences
	
	
## Pt 2. Identification of nuclear proteins	
	
4. Upload Step3_Proteomics.fasta and look for nuclear proteins, galaxy pipline pt2
	- Run on Galaxy: 
	Wolf PSort (plant), NLStradamus, Predict NLS and NoD ran to detect nuclear localisations
	- Concatenate lists, remove duplicate lines using count
	- Saved as 140716_Nucl-IDs.txt 2548 genes
	
5. Create Venn diagram, using Venny
	- Sequence IDs are copied from lists in Galaxy pipeline and entered in Venny software
	  Oliveros (2007)
	

## Pt 3. Data Filtering
	    
6. Detect Proteins that are Present/Absent between treatments
	- Use R script: Detect_PresenceAbsence.R
	requires Step6_proteinLFQs2.txt 
	- Creates 8 files proteins UP/DOWN by presence absence at 8 and 24 hour (e.g. between infected / non infected) or within a treatment (e.g. up or down in non infected from 8 to 24 hour)
	- Create Venn Diagram using protein names for each list.
										
